# Lipidomic Typing of Colorectal Cancer Tissue Containing Tumour-Infiltrating Lymphocytes by MALDI Mass Spectrometry Imaging

**DOI:** 10.3390/metabo11090599

**Published:** 2021-09-05

**Authors:** Vanna Denti, Allia Mahajneh, Giulia Capitoli, Francesca Clerici, Isabella Piga, Lisa Pagani, Clizia Chinello, Maddalena Maria Bolognesi, Giuseppe Paglia, Stefania Galimberti, Fulvio Magni, Andrew Smith

**Affiliations:** 1 Department of Medicine and Surgery, Proteomics and Metabolomics Unit, University of Milano-Bicocca, 20854 Vedano al Lambro, Italy; v.denti@campus.unimib.it (V.D.); allia.mahajneh@unimib.it (A.M.); francesca.clerici@unimib.it (F.C.); isabella.piga@unimib.it (I.P.); lisa.pagani@unimib.it (L.P.); clizia.chinello@unimib.it (C.C.); giuseppe.paglia@unimib.it (G.P.); fulvio.magni@unimib.it (F.M.); 2 Bicocca Bioinformatics Biostatistics and Bioimaging B4 Center, School of Medicine and Surgery, University of Milano-Bicocca, 20900 Monza, Italy; giulia.capitoli@unimib.it (G.C.) stefania.galimberti@unimib.it (S.G.); 3 Department of Medicine and Surgery, Anatomy and Pathology, University of Milano-Bicocca, 20900 Monza, Italy; maddalena.bolognesi@unimib.it

**Keywords:** MALDI-MSI, lipidomics, colorectal cancer, lymphocytes, immunity

## Abstract

Predicting the prognosis of colorectal cancer (CRC) patients remains challenging and a characterisation of the tumour immune environment represents one of the most crucial avenues when attempting to do so. For this reason, molecular approaches which are capable of classifying the immune environments associated with tumour infiltrating lymphocytes (TILs) are being readily investigated. In this proof of concept study, we aim to explore the feasibility of using spatial lipidomics by MALDI-MSI to distinguish CRC tissue based upon their TIL content. Formalin-fixed paraffin-embedded tissue from human thymus and tonsil was first analysed by MALDI-MSI to obtain a curated mass list from a pool of single positive T lymphocytes, whose putative identities were annotated using an LC-MS-based lipidomic approach. A CRC tissue microarray (TMA, *n* = 30) was then investigated to determine whether these cases could be distinguished based upon their TIL content in the tumour and its microenvironment. MALDI-MSI from the pool of mature T lymphocytes resulted in the generation of a curated mass list containing 18 annotated *m*/*z* features. Initially, subsets of T lymphocytes were then distinguished based on their state of maturation and differentiation in the human thymus and tonsil tissue. Then, when applied to a CRC TMA containing differing amounts of T lymphocyte infiltration, those cases with a high TIL content were distinguishable from those with a lower TIL content, especially within the tumour microenvironment, with three lipid signals being shown to have the greatest impact on this separation (*p* < 0.05). On the whole, this preliminary study represents a promising starting point and suggests that a lipidomics MALDI-MSI approach could be a promising tool for subtyping the diverse immune environments in CRC.

## 1. Introduction

There is an ever-increasing body of evidence to support the central role of the tumour microenvironment (TME) and its interactions with tumour infiltrating immune cells during the process of carcinogenesis [1]. This is particularly relevant in colorectal cancer (CRC), where those patients who progress to the later stages of the disease and present with distant metastasis, are often associated with unfavourable immune environments [2]. In fact, several studies have highlighted that an enhanced presence of certain tumour-infiltrating lymphocytes (TILs) may indicate an improved prognosis [3]. Despite this, the TNM staging system still remains the gold-standard classification system for colon cancer and, while this can be a powerful tool, it does not always provide complete prognostic information given that it does not consider this immune response of the host [4,5]. Moreover, without considering the immune status of a tumour, predicting responses to the diverse immunotherapy strategies also remains challenging [6,7].

For this reason, molecular approaches that are capable of classifying the immune environments associated with TIL infiltration are being readily investigated, with a particular focus on the proteome [8,9]. However, dysregulated lipid metabolism of tumour-infiltrating immune cells and their surrounding environment has also been demonstrated to be a driver of responsiveness and tolerance to immunosuppressive treatment [10], including in CRC [11]. Thus, studying the tissue lipidome of colorectal cancer may also provide an indication of the immune environment and provide further assistance in the stratification of these patients. To address this challenge, spatial lipidomics may represent an ideal tool for typing these diverse immune environments in situ given that the spatial relationship of those dysregulated lipids within the cellular network remains intact and can account for the innate molecular heterogeneity observed in CRC [12,13,14].

In this proof of principle study, we aim to explore the feasibility of using spatial lipidomics by MALDI-MSI to distinguish CRC tissue based upon their TIL content, using archived formalin-fixed paraffin-embedded (FFPE). To do so, we used a pool of single positive T lymphocytes from human thymus tissue to generate a curated mass list that was indicative of a lymphocyte rich environment. This was then applied to a CRC tissue micro array (TMA, *n* = 30) to determine whether these cases could be distinguished based upon their TIL content in the tumour and its microenvironment.

## 2. Results

### 2.1. Generating Lipid Signatures of Single Positive T Lymphocytes in Human Thymus and Tonsil Tissue

**Figure 1:**
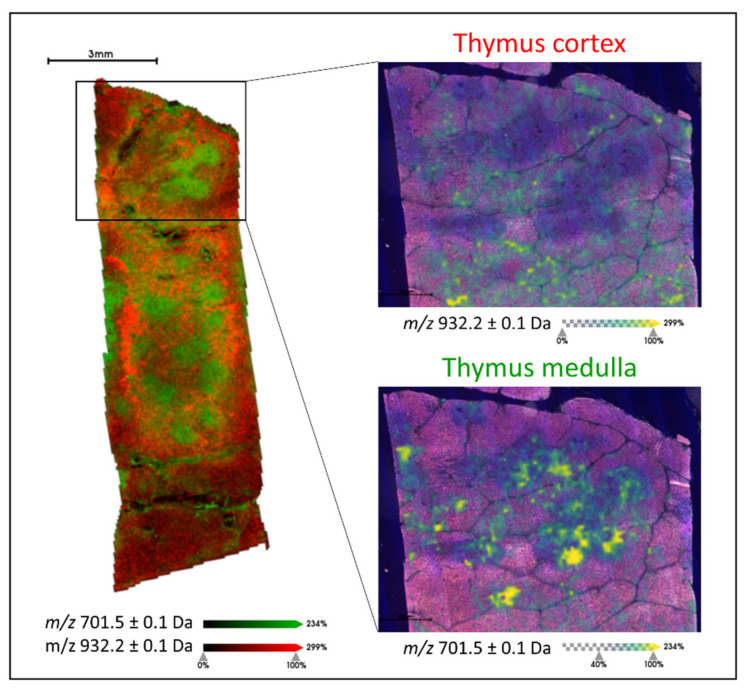
Example MALDI-MS image overlay of *m*/*z* 701.5 [PA(18:0/18:1)] (green) and *m*/*z* 932.2 (red) which are localised to the medulla and cortex regions of the thymus, respectively. The two insets present the individual ion images (green to yellow scale) overlaid with the haematoxylin and eosin (H&E) stained image of the tissue section, with the cortex and medulla characterised by the lighter and darker regions, respectively. Intensity and scale bars are provided.

Initially, the human thymus tissue was analysed by MALDI-MSI in order to generate a lipid signature (of *m*/*z* values) that was indicative of an environment rich in T lymphocytes. To achieve this, the medulla region of the thymus was considered given that it contains single positive T lymphocytes (CD4^+^ or CD8^+^, SP), in the later stages of maturation and differentiation, which may then be found in the colorectal cancer tissue. From those *m*/*z* signals that followed the morphology of the medulla region of the thymus and were detected as *m*/*z* features during the feature detection pipeline, a total of 18 could be annotated using liquid chromatography coupled with mass spectrometry (LC-MS)-based lipidomic approach and these are summarised in Supplementary Materials S1. The mass list obtained from this pool of single positive T lymphocytes was then employed to determine whether it could be used to differentiate them from those double-positive (DP) T lymphocytes in the earlier stages of maturation and differentiation. When comparing the lipid profiles of SP and DP T lymphocytes in thymus tissue, differences in their profiles were observed within the *m*/*z* 700 to 950 range (Supplementary Figure S1). In fact, of the 18 *m*/*z* features, four of them *m*/*z* 701.5 [PA(18:0/18:1)], 702.6 [PE(40:2)], 723.4 [PA(20:4/18:0)], and 725.4 [PA(18:0/18:3)] could discriminate SP from DP T lymphocytes. This is illustrated in Figure 1, where *m*/*z* 701.5 is overlaid with *m*/*z* 932.2, a lipid signal whose tissue distribution was localised to the cortex region of the thymus (DP T cells). The latter signal, *m*/*z* 932.2, was not detected as an *m*/*z* feature in the medulla due to its insufficient intensity and for this reason, it is not present in the curated peak list.

**Figure 2:**
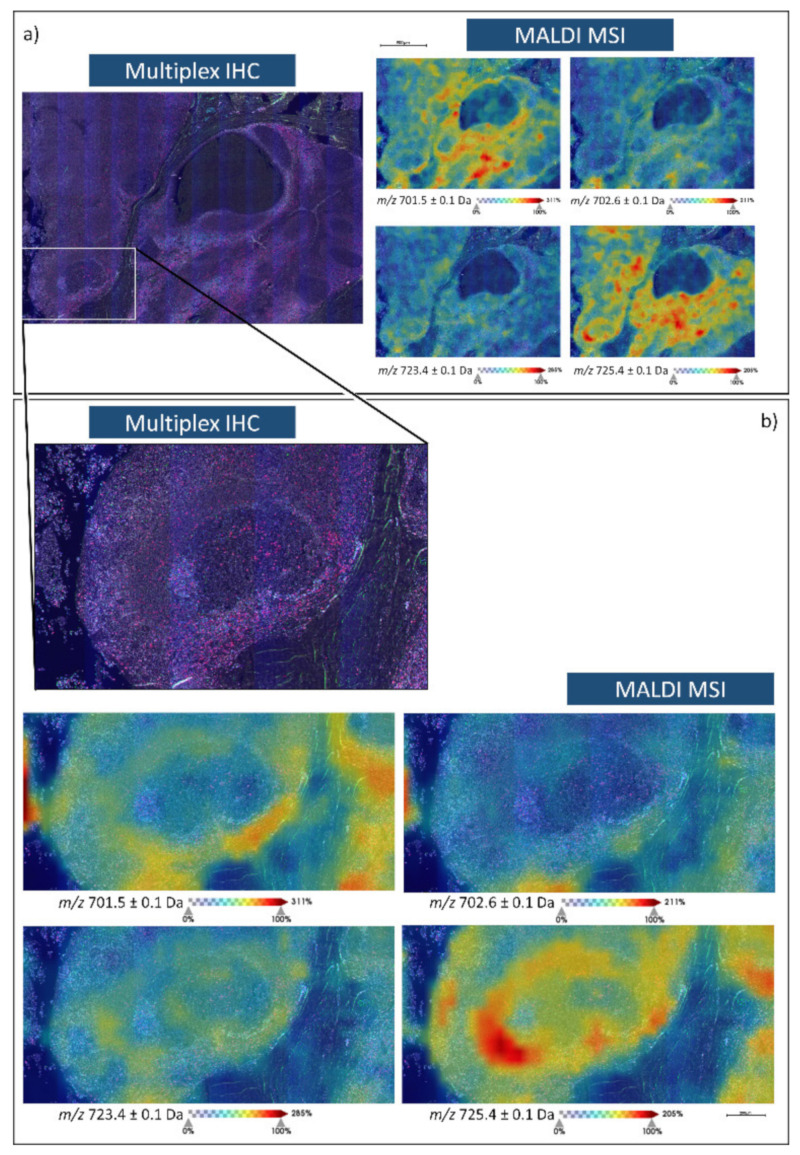
Multiplex immunohistochemistry (Blue, CD3; Green CD4; Red, CD8, Grey, 4′,6-diamidino-2-phenylindole (DAPI)) and MALDI-MS images of *m*/*z* 701. 5 [PA(18:0/18:1)], 702.6 [PE(40:2)], 723.4 [PA(20:4/18:0)], and 725.4 [PA(18:0/18:3)] in human tonsil tissue. (**a**) is representative of a larger area of the tonsil which encompasses both lymphocytes and epithelial cells whilst (**b**) provides an inset from a follicle that is surrounded by a denser distribution of T lymphocytes.

In order to confirm the robustness of this SP T lymphocyte lipid signature, the presence of these *m*/*z* lipid features was also evaluated in human tonsil tissue, which is also known to be a further site of SP T lymphocyte production. Here, multiplex immunohistochemistry (IHC) for CD3, CD4, and CD8 (T lymphocyte markers) was also performed on a consecutive tissue section in order to confirm the presence of these *m*/*z* features in those regions containing SP T lymphocytes. As exemplified in Figure 2, *m*/*z* 701.5 [PA(18:0/18:1)], 702.6 [PE(40:2)], 723.4 [PA(20:4/18:0)], and 725.4 [PA(18:0/18:3)] whose signal intensity could differentiate SP from DP T lymphocytes in thymus tissue, were all localised to regions of the tonsil with the greatest T lymphocyte content and were of much lower in the regions containing epithelial cells.

### 2.2. Distinguishing CRC Tissue Based upon Its Tumour Infiltrating Lymphocyte Content

**Figure 3:**
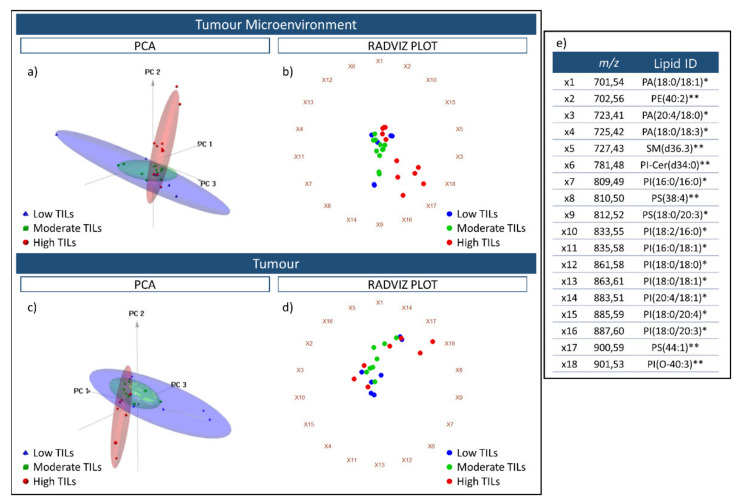
Three-dimensional PCA score charts of the Low TIL (blue), Moderate TIL (Green), and High TIL (red) groups using spectra obtained from the tumour microenvironment (**a**) and tumour (**c**) regions. The first three components explained 93.2% (PC1 74.3%; PC2 11.8%; PC3 6.2%) of the variability within the data. Radviz plots generated using the same spectra from the tumour microenvironment and tumour are presented in (**b**,**d**). The 18 *m*/*z* features that were used for the PCA analysis and Radviz plots are reported in (**e**) and are linked with the identifiers (ie. X1) that are placed around the circumference of the Radviz plot. The putative lipid ID assigned to these *m*/*z* features is also provided; * indicates those lipids annotated based on accurate mass, isotopic distribution and MS/MS whilst ** indicates those lipids annotated based on accurate mass and isotopic distribution.

Subsequently, a tissue microarray (TMA) consisting of 30 cores from colorectal cancer (CRC) cases was analysed by MALDI-MSI to determine the feasibility of distinguishing these tissues on the basis of their tumour infiltrating lymphocyte (TIL) content, with the cores being assigned to a Low (*n* = 10), Moderate (*n* = 10), or High TIL (*n* = 10) content group. When performing unsupervised PCA analysis using the spectra obtained from the tumour microenvironment (TME) and the tumour regions, respectively, those cases with a high TIL content were separated from the Low and Moderate TIL groups along the second component (Figure 3a,c), which explained 11.8% of the variability within the dataset after the first division, respectively. Moreover, when the cases were plotted using another multivariate data visualization algorithm, namely a Radviz plot, this general separation between the High TIL group and the Low and Moderate TIL group remained apparent, being slightly more evident in the TME than it was in the tumour region (Figure 3b,d). However, in both instances, this separation was influenced most heavily by three features, *m*/*z* 883.1 [PI(20:4/18:1)], *m*/*z* 900.6 [PS(44:1)], and *m*/*z* 901.5 [PI(O-40:3)].

To further investigate the possibility to discriminate among CRC cases with different levels of TIL content based upon their lipidomic profile, the Kruskal–Wallis test (two-sided, α = 0.05 with adjustment for multiplicity) was used to compare the intensity levels of the 18 lipid features among the three classes; Low, Moderate, and High TILs whilst a post-hoc Wilcoxon Rank Sum test was applied for pairwise comparisons. In particular, three of the features had a significant *p*-value in the TME, PI(20:4/18:1), PS(44:1), and PI(O-40:3) when comparing among the three groups, and were those previously highlighted in the Radviz plot. When comparing the High TIL group with the Low and Moderate TIL groups, respectively, using a pairwise comparison, *p*-values of 0.0046 [PI(20:4/18:1)], 0.0079 [PS(44:1)], and 0.0107 [PI(O-40:3)] were calculated. These values, along with the observed fold change for each of the 18 *m*/*z* features, are reported in Supplementary Materials S1.

**Figure 4:**
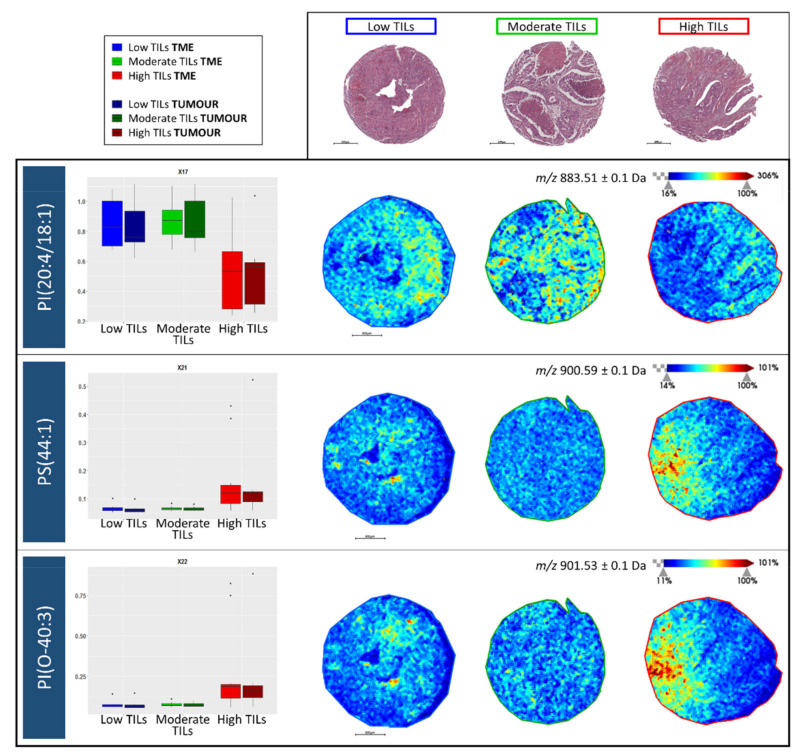
(**Left**) Intensity box plots for *m*/*z* 883.1 [PI(20:4/18:1)], *m*/*z* 900.6 [PS(44:1)], and *m*/*z* 901.5 [PI(O-40:3)] among the Low tumour infiltrating lymphocyte (TIL) (blue), Moderate TIL (green), and high TIL (red) groups in both the tumour microenvironment (TME) and tumour regions. (**Right**) Example MALDI-MS images illustrating the distribution of these three lipid features in colorectal cancer tissue cores. Intensity and scale bars are provided whilst the H&E stained images of the same cores, obtained after MALDI-MSI and liquid chromatography coupled with tandem mass spectrometry (LC-MS/MS) analysis, are provided above for reference.

When visualising the trend in intensity of PI(20:4/18:1), PS(44:1), and PI(O-40:3) in the TME as well as in tumour regions, opposite trends were observed among the three TIL groups. The intensity of PI(20:4/18:1) decreased significantly within the High TIL group whilst the intensity of PS(44:1) and PI(O-40:3) increased significantly in the High TIL group with respect to the others (Figure 4 Left). Moreover, this trend was also evident when visualising their tissue distributions, however, in the High TIL group, these two lipid features localised to different regions of the lymphocyte rich CRC tissue (Figure 4 Right).

### 2.3. Correlating Lipid Signal Distribution with CD3 and CD8 Immunohistochemistry

**Figure 5:**
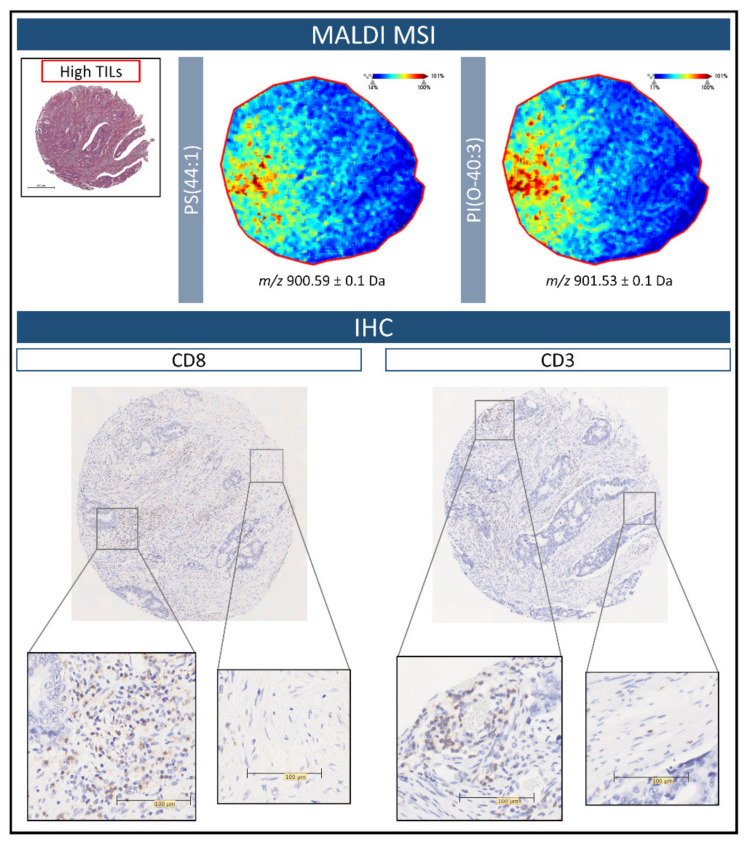
(**Top**) Example MALDI-MS images illustrating the distribution of *m*/*z* 900.6 [PS(44:1)], and *m*/*z* 901.5 [PI(O-40:3)] within colorectal cancer tissue of the High tumour infiltrating lymphocyte (TIL) group. The H&E stained image is provided in order to reference the overall tissue morphology. (**Bottom**) Immunohistochemistry images for CD3 and CD8 performed on further sections of the colorectal cancer tissue microarray. The greatest number of TILs, in particular CD8, are found within the regions highlighted by the MALDI-MS images. Zoomed insets have been provided in order to better visualize the presence of the TILs. Scale bars are provided.

In order to correlate the tissue distribution of those lipids detected in the High TIL group with the presence of lymphocytes, immunostaining for CD3 and CD8 (T lymphocyte markers) was performed on two additional sections of the colorectal cancer TMA. To exemplify these preliminary findings, the same tissue core representative of the High TIL group in Figure 4 was utilised. As presented in Figure 5 Top, the signals relative to PS(44:1) and PI(O-40:3) are both distributed throughout the left side of the tissue core. Upon viewing the IHC images for CD3 and CD8, these regions also contained the highest number of T lymphocytes, in particular CD8, and were distributed quite widely in these tissue regions. Moreover, in those regions with the lowest intensity of these two lipid signals, the TIL content was scarce.

## 3. Discussion

Predicting the prognosis of colorectal cancer (CRC) patients remains challenging, and while the traditional TNM staging system provides an excellent diagnostic overview, it often does not provide prognostic information or allow the response of patients to immune checkpoint inhibitors to be predicted [15,16]. To overcome these limitations, multiple means of performing subtyping of these cases are being investigated, with many approaches focusing on the presence of tumour infiltrating lymphocytes (TILs) given that an enhanced presence of certain TILs may indicate an improved prognosis and response to immunotherapy [17,18]. This may not be surprising considering that lymphocytic infiltration is a major immunological defence against tumour cells in solid tumours, especially in the microenvironment where inflammatory activity plays a key role in initiating an appropriate immune response [19,20]. In particular, several studies have shown that the number and type of TILs are prognostic indicators for disease-free survival in numerous malignancies, including CRC, and are also associated with the response of a tumour following treatment with traditional immunotherapy [21,22,23]. While immunohistochemistry (IHC)-based approaches are gathering steam for this purpose, they may also suffer from the general weaknesses associated with IHC [24,25] and, thus, approaches that target complementary molecular levels may provide adjunctive support.

In this proof of principle study, we employed MALDI-MSI to assess the feasibility of detecting lipidomic signatures that are associated with the presence of TILs in CRC tissue. It should be noted that this study was performed on clinical FFPE tissue, which represents the common archived tissue source in clinical centres, and is often problematic for spatial lipidomics studies using MALDI-MSI given that many lipids are depleted during the initial processing with organic solvents [26]. However, recent studies employing MALDI-MSI and FTIR spectroscopy, respectively, have provided evidence to suggest that some of these solvent-resistant lipid species are maintained in FFPE tissues and may provide diagnostically relevant information [26,27,28,29]. Nevertheless, we wanted to be sure that the low-intensity *m***/***z* features present in fact corresponded with tissue lipids, rather than chemical artefacts, such as 9-Aminoacridine adducts [30] that fall within the same mass range and may not be fully resolved with MALDI-TOF instrumentation and were more specific for a lymphocyte-rich environment. To achieve this, we generated a curated mass list from a pool of single positive T lymphocytes present within human thymus tissue, including only those features that could be assigned an identity using an LC-MS-based lipidomic approach.

Given that lipid metabolism has been demonstrated to have an enormous impact on the ability of T cells to properly activate and differentiate into effector or regulatory subsets [31,32], we first utilised this curated mass list of 18 *m***/***z* features, to differentiate subsets of thymocytes based on their level of differentiation, as presented in Figure 1 and Supplementary Figure S1. This was an intriguing starting point given that whilst only a restricted mass list was considered, the biological differences present within the thymus could still be explained, further supporting recent studies that suggest that some of the solvent-resistant lipid species maintained in FFPE tissues may provide biologically relevant information [26,29].

After verifying that these lipid signals were also present within human tonsil tissue, in particular in those regions with a high lymphocyte content (Figure 2), we then proceeded to assess the feasibility of distinguishing CRC tissue cores (*n* = 30) that were characterised by differing levels of TILs, considering the tumour microenvironment (TME) and tumour as independent histological features. As presented in Figure 3a,c, those cores with a high TIL content were well separated from those with a lower TIL content. However, the greatest division was observed within the TME (Figure 3b,d) and whilst this could be explained by molecular interactions that may occur between the TME and TILs [33], along with the higher number of TILs with respect to the tumour regions, we must also consider that a larger number of biological variables are present within the tumour [34], such as tumour stage, and this may have been a confounding factor considering the limited case series.

In particular, three *m***/***z* features, *m***/***z* 883.1 [PI(20:4/18:1)], *m***/***z* 900.6 [PS(44:1)], and *m***/***z* 901.5 [PI(O-40:3)] were shown to have the greatest impact when separating the High TIL cases from the Low and Moderate TIL cases, with a decreased and increased intensity, respectively, with this trend being particularly evident within the tumour microenvironment. In particular, phosphatidylinositol species play an essential role in a wide range of cellular functions such as ligand-receptor signal transduction and membrane trafficking. Alterations in the phospholipid composition of the plasma membrane have an impact on T lymphocytes functions and contribute to inflammatory responses or immune suppression [35]. Given that MALDI-MSI in negative ion mode is particularly adept at detecting this class of lipids, it may suggest why those cases with differing TIL content may be distinguished using this approach, especially considering that high TIL content is associated with an inflamed immune environment that also contains a greater breadth of T lymphocytes [36]. It is also interesting to note that in the cores with a high TIL content, PI(20:4/18:1), PS(44:1), and PI(O-40:3) were localised to different regions of histologically similar tissue. When performing preliminary correlations of the MALD-MSI data with IHC, the highest signal intensity for *m***/***z* 900.6 [PS(44:1)] and *m***/***z* 901.5 [PI(O-40:3] were found in regions of CRC tissue with a high number of CD3 and, in particular CD8, T lymphocytes (Figure 5), with a negligible intensity being observed in those regions with scarce TIL content. This may be suggestive of the different T lymphocytes and immune environments present in these cores, however, this should be confirmed in future studies using a greater number of T lymphocyte markers that also provide a more comprehensive indication of the tumour immune environment [19]. Moreover, this proof of principle study was performed on a relatively limited number of CRC cases, and a more in-depth study that considers a larger cohort and also incorporates the clinical outcome of these patients is required to confirm these preliminary findings and evaluate how spatial lipidomics could provide a complementary adjunct in this field.

## 4. Materials and Methods

### 4.1. Histopathology

The FFPE blocks selected for this study included human thymus and tonsil tissue as well as a series of 30 FFPE colorectal cancer tissue specimens that were collected from the archive of the Department of Pathology at the University of Milano Bicocca, Monza, Italy. The study was approved by the Institutional Review Board Comitato Etico Brianza, N. 3204, “High-dimensional single cell classification of pathology (HDSSCP)”, October 2019. Patient consent was obtained or waived according to article 89 of the EU General Data Protection Regulation 2016/679 and decree N.515 of the Italian Privacy Authority (19 December 2018).

Tissues were fixed in formalin for 24 h after surgical procedures and subsequently grossed and processed as routine cases; a representative histological haematoxylin and eosin (H&E) stained section of the original tumours was evaluated by an expert pathologist and the corresponding paraffin block was chosen for the TMA sampling. FFPE blocks were sampled using 2 mm diameter cores; areas with necrosis, inflammation or artefacts were carefully excluded. A total of 30 cores were transferred onto a TMA block. The TMA layout was built using the ISE Galileo TMA R4.30 software (Integrated Systems Engineering, Milan, Italy). The realization of the TMA blocks was made on a semi-automatic arrayer ISE Galileo TMA CK 4500 (Integrated Systems Engineering, Milan, Italy).

TNM staging data were available for 21 of the 30 CRC cases included in the TMA and are summarised as follows; pT1 (*n* = 3), pT2 (*n* = 5), pT3 (*n* = 8), and pT4 (*n* = 5), while metastasis (M) was not present in any of the cases. Moreover, immunohistochemistry data for MLH1 status were available and indicated that expression was retained in all of the 30 cases.

### 4.2. Sample Preparation for MALDI-MS Imaging

Four-micron-thick sections were cut and mounted onto conductive glasses coated with indium tin oxide (Bruker Daltonik GmbH, Bremen, Germany). Three consecutive tissue sections were obtained from the thymus, with the analysis performed in triplicate, while one was obtained from the colorectal cancer TMA. The slides were stocked at room temperature until the day of the analysis. The FFPE tissue sections were firstly deparaffinised [28] and allowed to dry on a heat bed at 30 °C. At this point, four fiducial marks were also placed on the ITO glass slide, surrounding the corners of the tissue containing region. Subsequently, 10 mg/mL 9-aminoacridine (9-AA) was dissolved in a 70% methanol solution and deposited using the HTX TM-Sprayer™ (HTX Technologies, LLC, Chapel Hill, NC, USA) with the following parameters: temperature 85 °C; number of passes 6; flow rate 0.2 mL/min; velocity 1100 mm/min; track spacing 2 mm; pressure 10 psi.

### 4.3. MALDI-MS Imaging

All imaging analyses were performed using a rapifleXTM MALDI Tissuetyper^TM^ mass spectrometer (Bruker Daltonics, Bremen, Germany) equipped with a Smartbeam™ 3D laser. External calibration was performed using red phosphorus clusters in the *m***/***z* range of 0 to 2000 [26]. Mass measurements were performed in negative ion reflectron mode in the *m***/***z* range of 500 to 900. The matrix suppression deflection was set to *m***/***z* 400 and the spectrometer voltages were set as following; Ion Source 1 = 19.93 kV, PIE = 2.58 kV, Lens = 10.95 kV, Reflector 1 = 20.87 kV, Reflector 2 = 0.97 kV, Reflector 3 = 8.76 kV.

For the MALDI-MS images acquired from the thymus and tonsil, 500 shots were accumulated for each spectrum and the measurement regions were rasterised at a lateral resolution 50 × 50 (x, y) µm with a laser scan range of 44 µm per pixel. For those acquired from the colorectal cancer tissue, 200 shots were accumulated for each spectrum and the measurement regions were rasterised at a lateral resolution 10 × 10 (x, y) µm with a laser scan range of 6 µm per pixel. Laser focus adjustment was performed for each line of the tissue microarray.

### 4.4. Data Pre-Processing

Data files containing the individual spectra of each entire measurement region were imported into SCiLS Lab MVS 2021c software (http://scils.de/; accessed on 15 July 2021, Bremen, Germany) for spectra processing and to transfer the annotated Regions of Interest (ROIs) indicated by the pathologist to the dataset. The processing steps included baseline subtraction (Convolution algorithm), normalisation (Total Ion Current algorithm), and spatial denoising (Weak). Average spectra, representative of the whole measurement regions and the ROIs, were generated to display differences in the lipid profiles.

For the human thymus dataset, the “Find Peaks” feature of the Segmentation Pipeline in SCiLS Lab MVS 2021c was used to generate an initial mass list from the medulla (SP T lymphocytes); this feature considers the signal-to-noise ratio, peak shape, and the number of spectra in which the signal is found in order to define whether it can be assigned as a peak. This mass list was then curated further using the LC-MS based lipidomics described in the following section.

### 4.5. LC-MS-Based Lipidomics

In order to obtain a lipid signature that was indicative of an environment rich in single positive (SP) T lymphocytes and to generate a curated peak list which could then be employed during all subsequent data processing, a target mass list containing those *m*/*z* features whose tissue localisation was in agreement with the morphology of the thymus medulla, and was present in all three of the technical replicates, was generated. To identify those *m*/*z* features present within this mass list, lipids were extracted from those thymus tissue sections previously analysed with MALDI-MSI. Firstly, the matrix was removed and collected using methanol and subsequently dried using an HETO vacuum concentrator. Subsequently, 100 µL of methanol was used in order to obtain a concentrated solution.

The LC-MS analysis was performed using an Agilent 1290 Infinity II LC coupled to a 6546 LC/Q-TOF system. Lipid separation was carried out with an ACQUITY UPLC^®^ BEH C18 1.7 µm, 2.1× 100 mm Column (Waters™) at 55 °C using a 10 min gradient. A final concentration of 10 mM ammonium acetate was added to both mobile phase A (acetonitrile:water:acetic acid 60:39:1) and B (Isopropanol). The gradient was set starting from 45% up to 99% of phase B over 8 min, held for 1 min before decreasing to 45% for another minute. MS was operating in target MS/MS mode, negative ion polarity. Samples were analysed in full scan mode and product ion mode, using a fixed collision energy of 30 eV. Lipids were identified by performing a database search using MassHunter Qualitative Analysis (Agilent) software and an Agilent Personal Compound Database and Library (PCDL), employing an MS tolerance of 5 ppm and an MS/MS tolerance of 10 ppm.

The *m*/*z* features detected in the target mass list were then annotated using the obtained lipid identification list: a lipid identification was putatively assigned to a feature if an error of less than ±0.1 Da was observed between the *m*/*z* value observed in MALDI-MSI and the theoretical *m*/*z* of the lipid species identified by LC-MS/MS. In instances where an identity could not be assigned based upon LC-MS/MS data, a putative lipid identity was assigned if an error of ±0.05 Da was observed between the *m*/*z* value observed in MALDI-MSI and the theoretical *m*/*z* of the lipid species annotated by the LC-MS data alone. This is reflected in the reporting of these putatively annotated lipids. Any *m*/*z* features that could not be annotated using one of these criteria were excluded from the mass list. This resulted in the annotation of 18 *m*/*z* features and this list is provided in Supplementary Materials S1. Supplementary Materials S2 provides an overview of those fragment ions used to identify 12 of the 18 lipid signals by MS/MS.

### 4.6. Histological Staining

Following MALDI-MS, and LC-MS/MS analysis in the instance of the thymus tissue, sections were washed with ethanol (70% and 100%) and the slides were stained using haematoxylin and eosin (H&E). The slides were converted to the digital format by scanning using a ScanScope CS digital scanner (Aperio, Park Center Dr., Vista, CA, USA). In the instance of the thymus tissue, the medulla and cortex regions, containing SP and double positive (DP) T lymphocytes, respectively, were annotated. In the instance of the colorectal cancer TMA, the immune environment of each core was defined by a pathologist (G.C.) based upon the number of tumour-infiltrating lymphocytes (TILs) present: Low TILs, Moderate TILs, and High TILs, with 10 tissue cores were assigned to each group. The lymphocyte content in tissue cores assigned to these categories is exemplified in Supplementary Figure S2. Subsequently, the tumour and tumour microenvironment (TME) regions were annotated. These regions excluded any tissue regions with formed sclerosis, fibrosis, or necrosis. These annotations were maintained for the data analysis in Section 4.7.

### 4.7. Immunohistochemistry for CD3, CD4, and CD8

For the IHC of human thymus and tonsil tissue, a multiplex IHC approach employing sequential immunostaining and antibody removal was performed as described in Bolognesi et al. [37]. Briefly, polyclonal rabbit anti-CD3 (Sigma Aldrich, C7930), monoclonal mouse recombinant anti-CD4 (Abcam, ab133616), and monoclonal mouse IgG2a κ anti-CD8-β (Santa Cruz Biotechnology, sc-25277) antibodies were utilised. Individual IHC images were acquired with the Hamamatsu Nanozoomer S60 scanner (Nikon, Campi Bisenzio, Italia) is equipped with an Olympus 20×/0.75 PlanSApo objective, a Fluorescence Imaging Module equipped with an L11600 mercury lamp (Hamamatsu, Roma, Italy), a linear ORCA-Flash 4.0 digital CMOS camera (Hamamatsu) and two six-position filter wheels, one for excitation, the other for emission filters, and a three-cube turret. The multiplex images were then prepared as also described in Bolognesi et al.

For IHC of the colorectal cancer TMA, single immunostainings with polyclonal rabbit anti-CD3 (Sigma Aldrich, C7930) and monoclonal (F-5) anti-CD8-β antibodies were performed on further sections and the images acquired with the Hamamatsu Nanozoomer S60 scanner) the setup described above.

### 4.8. Statistical Analysis

Regarding the human thymus dataset, Receiver Operating Characteristic (ROC) analysis and Wilcoxon Rank Sum Test were performed, with an Area Under the Curve (AUC) of ≥0.70 and *p*-value of ≤0.05, respectively, were required for a peak from the annotated peak list to be considered as statistically significant when comparing environments containing single positive and double positive T lymphocytes. All these analyses were performed in SCiLS Lab MVS 2021c software.

Regarding the colorectal cancer dataset, the intensity level for each of the 18 *m*/*z* features included in the annotated mass list was extracted from the mean spectra from each core in the TMA. Using these values, unsupervised learning Principal Component Analysis (PCA) was performed to qualitatively assess the profile similarity among the three classes, Low, Moderate, and High TILs, in both the Tumour and TME region. Before the PCA analysis, data were scaled and centred. In the three-dimensional PCA score plot, the three components reported are those that were extracted from the PCA as the ones that explained the maximum variance of the original independent variables. Similarly, a Radviz plot was computed to visualise the multidimensional data set and determine which of the *m*/*z* features had the greatest impact. Finally, the non-parametric Kruskal–Wallis test with Benjamini-Hochberg adjustment (two-sided, α = 0.05) was used to compare the intensity levels of the 18 lipid features among the three classes: Low, Moderate, and High TILs. A post-hoc Wilcoxon Rank Sum test was applied for pairwise comparisons (Benjamini-Hochberg adjustment). All these analyses were performed in R software v.3.6.0.

## 5. Conclusions

In this proof of principle study, we employed MALDI-MSI to assess the feasibility of detecting lipidomic signatures that are associated with the presence of tumour infiltrating lymphocytes (TILs) in colorectal cancer tissue. Whilst many of the lipid species are depleted in formalin-fixed paraffin-embedded tissue, the small number that do remain may provide some biological information and were able to distinguish mature, differentiated, T lymphocytes from their immature counterparts within thymus tissue. Moreover, when this approach was applied to CRC tissue containing differing amounts of T lymphocyte infiltration, those cases with a high TIL content were distinguishable from those with a lower TIL content, suggesting the feasibility of this approach even in clinically archived tissue. As such, the proposed MALDI-MSI approach represents a promising tool for subtyping the diverse immune environments in CRC tissue that should be further investigated in a larger cases series.

## Data Availability

The data that support the findings of this study are available from the corresponding author, A.S., upon reasonable request.

## Supplementary Materials

The following are available online at https://www.mdpi.com/article/10.3390/metabo11090599/s1, Supplementary Materials S1: Summary of the curated mass list containing the m/z value, retention time obtained from liquid chromatography coupled with mass spectrometry (LC-MS), assigned lipid ID, and p-values/fold change obtained from the comparison of Low, Moderate, and High TIL groups in CRC tissue; Figure S1: Average mass spectra generated from the cortex (containing immature T lymphocytes; Red) and medulla (containing mature T lymphocytes; Green) regions of the thymus. These are presented in the m/z 700 to 950 range. Intensity is expressed as arbitrary units (a.u.). Asterisks indicate those m/z features presented in the ion images; Supplementary Materials S2: Overview of the fragment ions used to identify 12 of the 18 lipids within the curated mass list by liquid chromatography coupled with tandem mass spectrometry (LC-MS/MS); Figure S2: Overview of the lymphocyte content in the Low, Moderate, and High TIL groups of the CRC TMA, providing an overview of the histology (H&E) and CD3 IHC.
