# Supplementary figures and images for: Lipidomic Typing of Colorectal Cancer Tissue Containing Tumour-Infiltrating Lymphocytes by MALDI Mass Spectrometry Imaging
Source: Metabolites. 2021 Sep 5;11(9):599. doi: 10.3390/metabo11090599 (PMC8471593; doi:10.3390/metabo11090599)

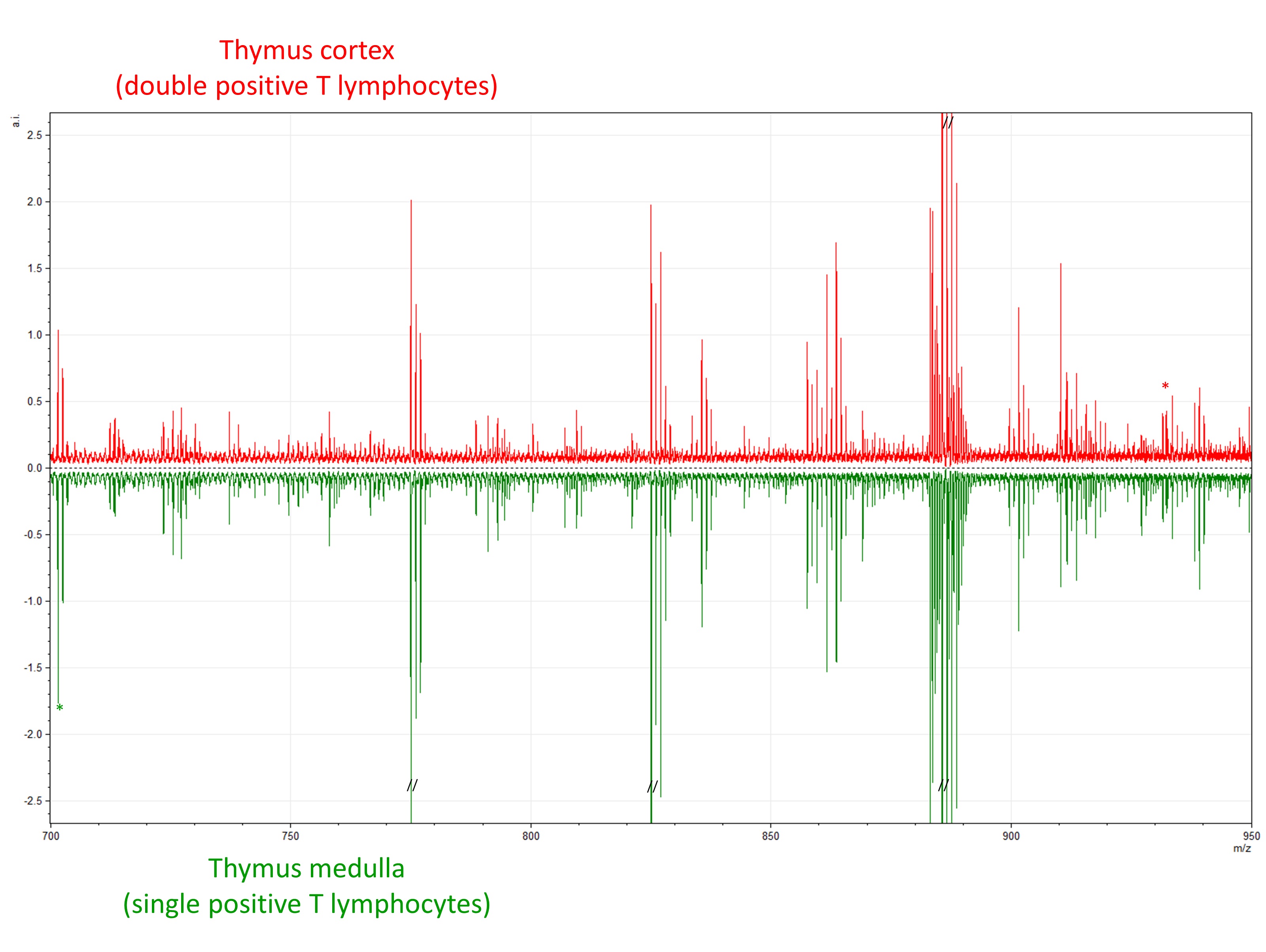

Supplement: Supplementary file 1 [file metabolites-11-00599-s001.zip › Supplementary Figure S1.jpg]

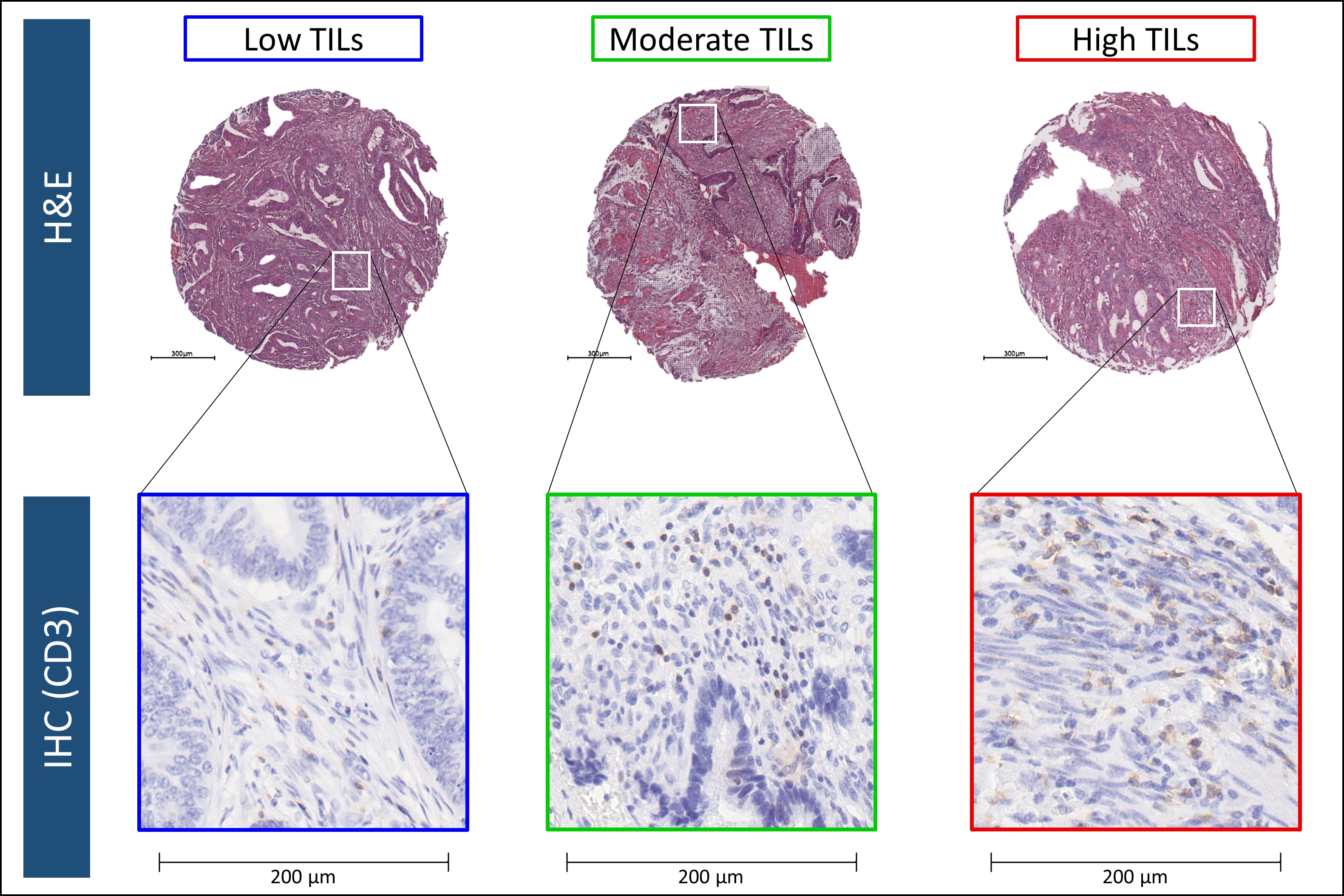

Supplement: Supplementary file 1 [file metabolites-11-00599-s001.zip › Supplementary Figure S2.jpg]
